# Supplementary figures and images for: Impact of COVID-19 vaccination on preventive behavior: The importance of confounder adjustment in observational studies
Source: PLoS One. 2024 Nov 25;19(11):e0313117. doi: 10.1371/journal.pone.0313117 (PMC11588266; doi:10.1371/journal.pone.0313117)

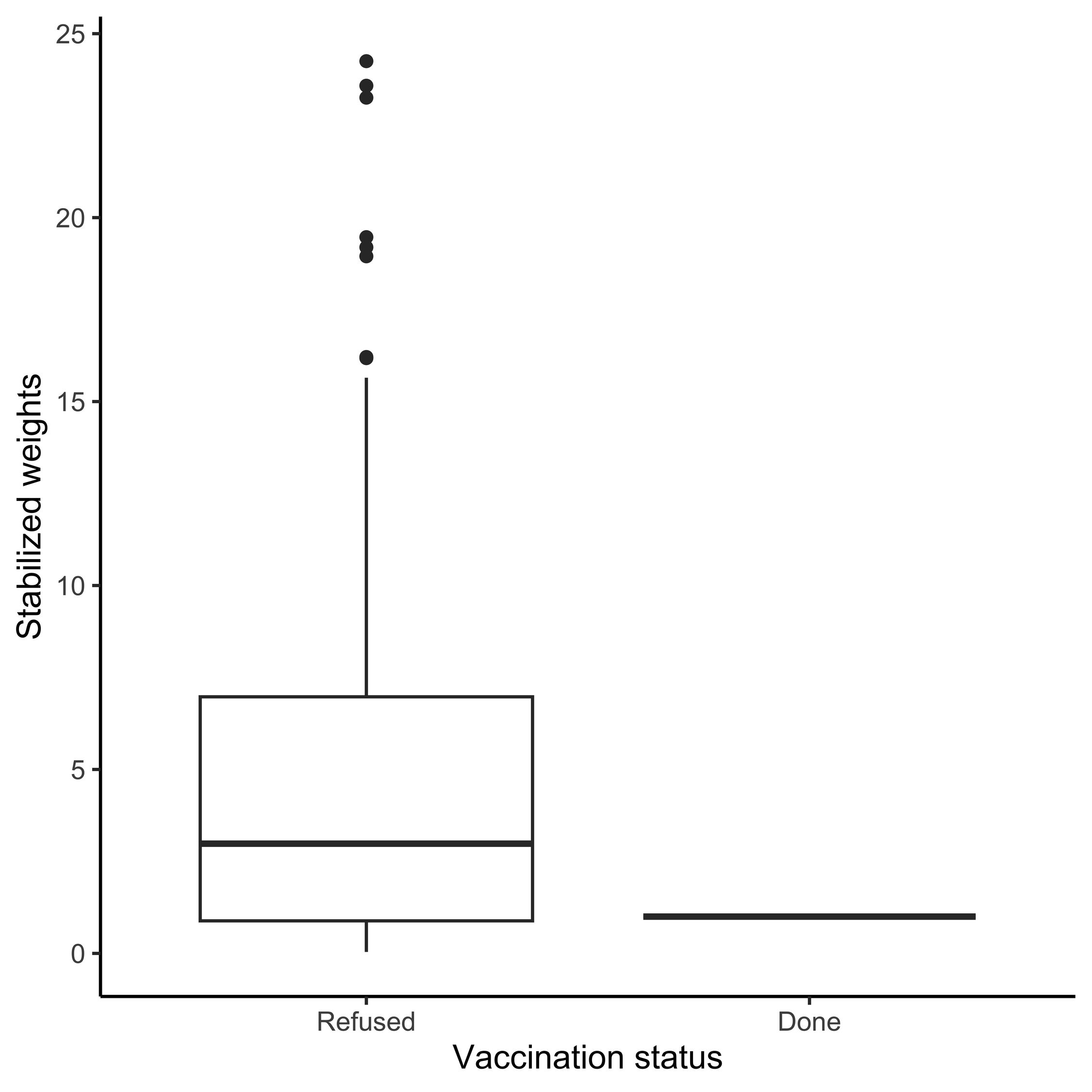

Supplement: S1 Fig — (TIF) [file pone.0313117.s001.tif]
