# Supplementary material for: Impact of COVID-19 vaccination on preventive behavior: The importance of confounder adjustment in observational studies
Source: PLoS One. 2024 Nov 25;19(11):e0313117. doi: 10.1371/journal.pone.0313117 (PMC11588266; doi:10.1371/journal.pone.0313117)
Supplement: Attachment — Submitted filename: 20240305_Review_PONE-D-24-03437.pdf [file pone.0313117.s002.pdf]

Review for PLOS ONE, manuscript PONE-D-24-03437

Biased Findings without Adjusting Methods in Observational Studies: Can Vaccination Status Influence Uptake of Preventive Measures against COVID-19?

The manuscript compares the bias of the ANCOVA method, and three versions of inverse probability of treatment weighted (IPTW) methods for the estimation of the average causal effect of a point-treatment on an outcome, and demonstrates all four methods using an empirical example concerning the effect of COVID-19 vaccinations on adherence to preventive measures. Personally, I am very much in favor of promoting methods that can adjust for large sets of covariates, as confounding seems to be a major is issue in (psychological, and social science) research that aims to answer causal questions (sometimes implicitly so) and using nonexperimental data. In that sense I support the author's overarching goal with this manuscript. At the same time, after having finished reading the manuscript, I do have a major concern, which I will elaborate on below. Following, I will elaborate on other concerns/questions that I had. I hope the authors find these comments useful for the improvement of the manuscript.

My major concern is that I was unsure about the "novelty" or "added-value" of the manuscript in relation to the vast amount of literature that already exists about this topic. As a didactical paper about PS-based methods, the manuscript falls short as it skips over many of the important details. While the authors do refer to other literature for the interested reader, such as Schafer and Kang (2008), I believe the manuscript does not offer any new insightful way of explaining the methods compared to the vast introductory literature that already exists and that goes into great detail (cf. Hernán and Robins, 2021; Naimi, Cole and Kennedy, 2016; Vansteelandt and Sjolander, 2016). As a methodological paper, the simulations are too simplified and limiting to provide any new insight into the performance of these methods in particular scenarios. The conclusion that unadjusted methods lead to bias in the presence of confounding, and PS-based methods (if the PS-model is correctly specified) not, is not new, and neither is the conclusion that IPTW-based methods can be incredibly inefficient (Robins, Hernán, Brumback, 2001). As an applied paper, the manuscript also falls short of many of the considerations and decisions researchers must make in practice (e.g., evaluation of post-balancing imbalance, evaluation of plausibility of conditional exchangeability and consistency assumptions). For improving the manuscript, I would advice the authors to make more explicit what this paper contributes to the existing literature.

A second concern is the treatment of selection bias in the manuscript. It is mentioned multiple times in the manuscript (e.g., lines 78, 134), and it plays a role in the simulation study (see Section 3.2). However, it is unclear to me what point the researchers are trying to convey with this, as the primary focus in the introduction, discussion and conclusion is about confounding bias.

Some (relatively) minor comments are mentioned below:

1. In line 55, there is mention of "non-probability samples". How does this term relate to the rest of the introduction, in which the focus is predominantly on confounding bias? Is "non-probability sample" a means of saying that the treatment is not randomized, or do the authors refer to selection bias here?

2. In line 57, the authors mention that "The use of regression models is preferred for large samples because (…)". Is this true *generally*? Schafer and Kang (2008) state "[PS-based] methods are in part a reaction to misapplications of ANCOVA, where analysts were often unaware of the sensitivity of their results to model failure.". At the least, such a statement seems unnuanced, and there are definitely situations with large samples in which PS-based methods are preferred.

3. Line 70 mentions that use of propensity scores can improve precision. This seems unnuanced, as IPTW-based methods are known to be inefficient (Vansteelandt and Joffe, 2014).

4. The last two sentences of this paragraph (lines 71-74) seem rather unrelated to the introduction of multiple PS-based methods, and read a bit weirdly. Personally, I would remove them, as these techniques from econometrics play no further role in this manuscript.

5. Line 77 is the first line that introduces IPTW. Given that this plays a major role in the simulations, I would introduce it earlier, rather than mentioning PS-methods generally.

6. Lines 102-103, this sentence is unclear to me.

7. Lines 108-111 mentions parametric assumptions for the ANCOVA method to work well. However, this seems nuanced again: You can also specify nonlinear outcome models with interactions. When this model is correct, then the coefficient of the binary exposure can be interpreted as a causal effect estimate. Furthermore, homoscedasticity is, I believe, not an issue for the bias of point estimates, but only for unbiased estimation of the standard errors (but please check me on this).

8. Lines 123-127 mentions causal identification assumptions for the PS-methods. However, these methods must equally hold for ANCOVA-methods (with ANCOVA, you also need no unmeasured confounders, for example). Similarly, the parametric assumptions mentioned for the ANCOVA method equally apply for PS-based methods, but now the PS-model needs to be correctly specified.

9. Line 134, Stabilized weights are not used because they reduce selection bias, right? Rather, because they reduce the variance of the weights and hopefully also improve precision of the causal effect estimates.

10. The Equation on line 167 includes Cohen’s d. What is the added value of using Cohen’s d here rather than simply picking some reasonable population value?

11. What is the reasoning for using B-splines, especially given the simple linear relationships in the generated data? I understand that in practice you don’t know if the true relationships are indeed linear, but the use of B-splines comes a little of the blue here.

12. For the results in Tables 1 and 2, what are the Monte Carlo SE’s of the bias estimates here (see Morris et al., 2016, for definition of this performance measure)? Because, in these results, the estimated bias for the scenario with n = 4000 and an effect of 2 (0.2 * d), the bias of the PS-based method is still approximately -0.2. This seems far off, given that both the outcome model and the PS-model should be able to rightly capture the linear relationships in the generated data.

13. In line 228, it is mentioned that “(…) with the estimated bias increasing with effect size (…)”. Indeed, a larger effect means more variance in the outcome, and logically speaking also a larger bias (right?). Therefore, wouldn’t it be better to report relative bias, rather than absolute bias (see Morris et al., 2016).

14. In line 235 you interpret estimates for gamma. Why would you want to interpret this parameter if your research question is only about the effect of X on Y? I think that most causal inference literature urges researcher not to interpret the parameters of confounders as these might be biased themselves, and the inclusion of these covariates only serves to adjust for confounding for the specific target parameter.

15. In Table 3, why use p-values? If you are assessing covariate balance, usually metrics like standardized mean differences (SMDs) are reported. Or if you are just describing sample characteristics, than p-values are not needed because you are not attempting to generalize to the population (i.e., there is no uncertainty about the characteristics of the people in the sample, as everyone in the sample was actually observed).

16. In Table 3, the outcome measures are also included. Are these actually end-of-study outcomes, or are these outcomes measured as earlier waves (and thus serve as confounders for the relationship between treatment and end-of-study outcome).

17. Line 293, please evaluate if weighting was successful by assessing, for example, SMDs in the balanced sample.

18. Line 296, how are these CI's computed? Based on robust SEs, bootstrap, or regular SEs?

19. Line 331, you mention "For a subset of respondents who reported being vaccinated at the beginning of (…)". This isn't the target population, right? It is those that were invited. Also, those that were invited might be a particular subpopulation of most vulnerable people. How does this influence the external validity of the results?

20. Empirical example generally, a serious treatment of the empirical example also warrants a serious evaluation of the causal identification assumptions that underlie the methods employed here. This is currently lacking. Do the authors think that the assumptions of exchangeability, consistency, positivity, and no interference (seems important in the context of vaccines) are plausible?
