# Supplementary material for: Impact of COVID-19 vaccination on preventive behavior: The importance of confounder adjustment in observational studies
Source: PLoS One. 2024 Nov 25;19(11):e0313117. doi: 10.1371/journal.pone.0313117 (PMC11588266; doi:10.1371/journal.pone.0313117)
Supplement: Attachment — Submitted filename: PONE-D-24-03437_ Review comments.pdf [file pone.0313117.s003.pdf]

Review comments: Biased Findings without Adjusting Methods in Observational Studies: Can Vaccination Status Influence Uptake of Preventive Measures against COVID-19?

Dear Editor,

This paper contributes to further understanding of the use of adjusting methods in observational studies. However, the key concern is that the distribution for the outcome used for simulation study is different from the one used for observational data. This makes the findings obtained from simulation studies less relevant to the observation study. Therefore, I recommend author to undertake simulation studies where the distribution of outcome variable is the same as the one used for observational studies. They also need to consider a situation when those assumptions are not met and evaluate how the results change. Considering this is a simulation study authors should share their analysis codes or links to enable study reproducibility, and this will help when paper is being reviewed.

I therefore recommend, major revision for the manuscript.

Major comments

1) Authors have clearly demonstrated statistical approach used for simulation studies in which the outcome was continuous. However, in the observational analysis they have used cumulative logistic model due to ordinal nature of the adherence to protective behaviour. I recommend that authors use identical distribution for both simulation and observational analyses. It would be appropriate to also consider undertaking sensitivity analysis when the linearity assumptions are not met. Considering the nature of the study, distribution used for simulation is supposed to the same as one applied using the data and this is not supposed to be included as a limitation. The analyses codes for both simulation and observational analyses should be made available for reproducibility purposes.

2) Authors need to present a DAGs for the COVID-19 observation study.

3) Make it clear whether survey weights were included in the PS model when computing weights?

Minor comments

Page 11 lines 63 -64 are not clear. Was it supposed to read "…characteristics that influence both the probability of the exposure and the outcome"?

In Table 3, summary of outcome variables (i.e. adherence to protective behaviour) by vaccination are not presented.

Page 26 line 347. State in which circumstances is PS advantageous.
