# Supplementary material for: Impact of COVID-19 vaccination on preventive behavior: The importance of confounder adjustment in observational studies
Source: PLoS One. 2024 Nov 25;19(11):e0313117. doi: 10.1371/journal.pone.0313117 (PMC11588266; doi:10.1371/journal.pone.0313117)
Supplement: Attachment — Submitted filename: reviewers_reply_def.docx [file pone.0313117.s004.docx]

**Reviewer 1**

The manuscript compares the bias of the ANCOVA method, and three versions of inverse probability of treatment weighted (IPTW) methods for the estimation of the average causal effect of a point-treatment on an outcome, and demonstrates all four methods using an empirical example concerning the effect of COVID-19 vaccinations on adherence to preventive measures. Personally, I am very much in favor of promoting methods that can adjust for large sets of covariates, as confounding seems to be a major is issue in (psychological, and social science) research that aims to answer causal questions (sometimes implicitly so) and using nonexperimental data. In that sense I support the author’s overarching goal with this manuscript. At the same time, after having finished reading the manuscript, I do have a major concern, which I will elaborate on below. Following, I will elaborate on other concerns/questions that I had. I hope the authors find these comments useful for the improvement of the manuscript.

My major concern is that I was unsure about the “novelty” or “added-value” of the manuscript in relation to the vast amount of literature that already exists about this topic. As a didactical paper about PS-based methods, the manuscript falls short as it skips over many of the important details. While the authors do refer to other literature for the interested reader, such as Schafer and Kang (2008), I believe the manuscript does not offer any new insightful way of explaining the methods compared to the vast introductory literature that already exists and that goes into great detail (cf. Hernán and Robins, 2021; Naimi, Cole and Kennedy, 2016; Vansteelandt and Sjolander, 2016). As a methodological paper, the simulations are too simplified and limiting to provide any new insight into the performance of these methods in particular scenarios. The conclusion that unadjusted methods lead to bias in the presence of confounding, and PS-based methods (if the PS-model is correctly specified) not, is not new, and neither is the conclusion that IPTW-based methods can be incredibly inefficient (Robins, Hernán, Brumback, 2001). As an applied paper, the manuscript also falls short of many of the considerations and decisions researchers must make in practice (e.g., evaluation of post-balancing imbalance, evaluation of plausibility of conditional exchangeability and consistency assumptions). For improving the manuscript, I would advice the authors to make more explicit what this paper contributes to the existing literature.

A second concern is the treatment of selection bias in the manuscript. It is mentioned multiple times in the manuscript (e.g., lines 78, 134), and it plays a role in the simulation study (see Section 3.2). However, it is unclear to me what point the researchers are trying to convey with this, as the primary focus in the introduction, discussion and conclusion is about confounding bias.

**Authors: We thank the reviewer for the comments and the opportunity to improve the manuscript. The manuscript has undergone a complete revision. We have updated the introduction to include recent findings from the literature. The simulation section has been replaced with a new one that uses the same generative model (cumulative logistic regression) as the real data application, thus offering the opportunity to add to the literature a simulation result that is commonly seen in real data applications in social sciences and psychology where the dependent variable is measured with a Likert scale. The simulation study now consists of two scenarios, depending on the absence/presence of model misspecification. Finally, we have removed the part related to bias selection from both the simulation and the discussion, as the main focus of the paper is on confounding bias. We hope you appreciate the changes made to the manuscript.**

Some (relatively) minor comments are mentioned below:

1. In line 55, there is mention of “non-probability samples”. How does this term relate to the rest

of the introduction, in which the focus is predominantly on confounding bias? Is “non-probability sample” a means of saying that the treatment is not randomized, or do the authors refer to selection bias here?

**Authors: Any reference to bias selection has been removed (also from the new simulation section) focusing on the confounding bias.**

2. In line 57, the authors mention that “The use of regression models is preferred for large samples because (...)”. Is this true *generally*? Schafer and Kang (2008) state “[PS-based] methods are in part a reaction to misapplications of ANCOVA, where analysts were often unaware of the sensitivity of their results to model failure.”. At the least, such a statement seems unnuanced, and there are definitely situations with large samples in which PS-based methods are preferred.

**Authors: We have better addressed this point specifying the situation in which covariate adjustment may fail.**

3. Line 70 mentions that use of propensity scores can improve precision. This seems unnuanced, as IPTW-based methods are known to be inefficient (Vansteelandt and Joffe, 2014).

**Authors: We have rephrased the sentence. In addition, the introduction section has been revised in order to explain in a preliminary way pros and cons of PS methods.**

4. The last two sentences of this paragraph (lines 71-74) seem rather unrelated to the introduction of multiple PS-based methods, and read a bit weirdly. Personally, I would remove them, as these techniques from econometrics play no further role in this manuscript.

**Author: We have removed these two sentences from the manuscript.**

5. Line 77 is the first line that introduces IPTW. Given that this plays a major role in the simulations, I would introduce it earlier, rather than mentioning PS-methods generally.

**Authors: We thank the Reviewer and in line with the comment, we have introduced earlier the IPTW method in the text with some additional sentences and references.**

6. Lines 102-103, this sentence is unclear to me.

**Authors: The sentence has been removed.**

7. Lines 108-111 mentions parametric assumptions for the ANCOVA method to work well. However, this seems nuanced again: You can also specify nonlinear outcome models with interactions. When this model is correct, then the coefficient of the binary exposure can be interpreted as a causal effect estimate. Furthermore, homoscedasticity is, I believe, not an issue for the bias of point estimates, but only for unbiased estimation of the standard errors (but please check me on this).

**Authors: We have now clarified the sentence.**

8. Lines 123-127 mentions causal identification assumptions for the PS-methods. However, these methods must equally hold for ANCOVA-methods (with ANCOVA, you also need no unmeasured confounders, for example). Similarly, the parametric assumptions mentioned for the ANCOVA method equally apply for PS-based methods, but now the PS-model needs to be correctly specified.

**Authors: We thank for the comment. We have modified the sentence accordingly.**

9. Line 134, Stabilized weights are not used because they reduce selection bias, right? Rather, because they reduce the variance of the weights and hopefully also improve precision of the causal effect estimates.

**Authors: Yes, we have added a short sentence justifying the use of stabilized weights.**

10. The Equation on line 167 includes Cohen’s d. What is the added value of using Cohen’s d here rather than simply picking some reasonable population value?

**Authors: We agree with the comment. In the new version of the results, the mention to the d of Cohen has been removed.**

11. What is the reasoning for using B-splines, especially given the simple linear relationships in the generated data? I understand that in practice you don’t know if the true relationships are indeed linear, but the use of B-splines comes a little of the blue here.

**Authors: We agree with the comment. In the new version of the manuscript, we use the Generalized Boosted Models, that is more commonly used for PS estimation than logistic regression.**

12. For the results in Tables 1 and 2, what are the Monte Carlo SE’s of the bias estimates here (see Morris et al., 2016, for definition of this performance measure)? Because, in these results, the estimated bias for the scenario with n = 4000 and an effect of 2 (0.2 * d), the bias of the PS-based method is still approximately -0.2. This seems far off, given that both the outcome model and the PS-model should be able to rightly capture the linear relationships in the generated data.

**Authors: The current results (now based on the average relative bias) have changed and they appear more in line with those present in literature.**

13. In line 228, it is mentioned that “(...) with the estimated bias increasing with effect size (...)”. Indeed, a larger effect means more variance in the outcome, and logically speaking also a larger bias (right?). Therefore, wouldn’t it be better to report relative bias, rather than absolute bias (see Morris et al., 2016).

**Authors: The new tables are now based on average relative bias, a measure that permits appropriate comparisons.**

14. In line 235 you interpret estimates for gamma. Why would you want to interpret this parameter if your research question is only about the effect of X on Y? I think that most causal inference literature urges researcher not to interpret the parameters of confounders as these might be biased themselves, and the inclusion of these covariates only serves to adjust for confounding for the specific target parameter.

**Authors: We have removed the confounder-to-outcome coefficient (gamma) from the tables.**

15. In Table 3, why use p-values? If you are assessing covariate balance, usually metrics like standardized mean differences (SMDs) are reported. Or if you are just describing sample characteristics, than p-values are not needed because you are not attempting to generalize to the population (i.e., there is no uncertainty about the characteristics of the people in the sample, as everyone in the sample was actually observed).

**Authors: Table 3 has been modified including the SMDs. As a consequence, the results section has been modified accordingly.**

16. In Table 3, the outcome measures are also included. Are these actually end-of-study outcomes, or are these outcomes measured as earlier waves (and thus serve as confounders for the relationship between treatment and end-of-study outcome).

**Authors: We are in presence of an observational study so both outcome, vaccination status and confounders were all asked at the same time. However, while the vaccination status reflected a past condition, the outcomes are asked as the current adherence to protective measures. Several studies investigated the effect of vaccination on protective behaviors because it was legitimate to hypothesize that risk compensation, in terms of personal safety related to vaccination, was offset by an increase in risky behaviors, such as socializing, traveling, and working outside the home.**

**Some studies analyzed the effect of vaccination between late 2020 and early 2021, the initial period of vaccine distribution, but did not find a change in adherence to protective behaviors (Wright et al., 2022; Desrichard et al., 2022) and justified the results as related to continued communication about the severity of the pandemic and the importance of barrier measures. Studies carried out in the following months (June 2021), showed instead a reduction in protective behaviors, underlined a weakened in people personal protective behavior after vaccination (Zhang et al., 2021).**

***Desrichard O, Moussaoui L, Ofosu N. Reduction of Precautionary Behaviour following Vaccination against COVID-19: A Test on a British Cohort. Vaccines. 2022; 10(6):936.*** [***https://doi.org/10.3390/vaccines10060936***](https://doi.org/10.3390/vaccines10060936)

***Wright, L.; Steptoe, A.; Mak, H.W.; Fancourt, D. Do People Reduce Compliance with COVID-19 Guidelines Following Vaccination? A Longitudinal Analysis of Matched UK Adults. J. Epidemiol. Community Health 2022, 76, 109–115.***

17. Line 293, please evaluate if weighting was successful by assessing, for example, SMDs in the

balanced sample.

**Authors: We have added a loveplot diagram in the supplementary material (Figure S1) to assess the ability of the SMDs in the balance sample.**

18. Line 296, how are these CI’s computed? Based on robust SEs, bootstrap, or regular SEs?

**Authors: These 95% CI were computed using standard SEs. We have added a word specifying they are based on regular Wald SEs.**

19. Line 331, you mention “For a subset of respondents who reported being vaccinated at the beginning of (...)”. This isn’t the target population, right? It is those that were invited. Also, those that were invited might be a particular subpopulation of most vulnerable people. How does this influence the external validity of the results?

**Authors: We believe that is an important point. The initial population of the COVID-19 vaccination campaign is a particular population of fragile people and essential workers (health workers, public officers, etc…). This particular selection may limit the external validity of the results and then the generalization of the reported findings. We have included a new paragraph in the discussion section.**

20. Empirical example generally, a serious treatment of the empirical example also warrants a serious evaluation of the causal identification assumptions that underlie the methods employed here. This is currently lacking. Do the authors think that the assumptions of exchangeability, consistency, positivity, and no interference (seems important in the context of vaccines) are plausible?

**Authors: In the results section we presented new results to assess exchangeability using PS methods, and in the discussion we have added a new paragraph considering the suggested issues in the context of observational studies and vaccines.**

**Reviewer 2**

Review comments: Biased Findings without Adjusting Methods in Observational Studies: Can Vaccination Status Influence Uptake of Preventive Measures against COVID-19?

Dear Editor,

This paper contributes to further understanding of the use of adjusting methods in observational studies. However, the key concern is that the distribution for the outcome used for simulation study is different from the one used for observational data. This makes the findings obtained from simulation studies less relevant to the observation study. Therefore, I recommend author to undertake simulation studies where the distribution of outcome variable is the same as the one used for observational studies. They also need to consider a situation when those assumptions are not met and evaluate how the results change. Considering this is a simulation study authors should share their analysis codes or links to enable study reproducibility, and this will help when paper is being reviewed. I therefore recommend, major revision for the manuscript.

**Authors: We thank for the comments. According to your suggestions and to those of Reviewer 1, the paper has completely revised.**

Major comments

1) Authors have clearly demonstrated statistical approach used for simulation studies in which the outcome was continuous. However, in the observational analysis they have used cumulative logistic model due to ordinal nature of the adherence to protective behaviour. I recommend that authors use identical distribution for both simulation and observational analyses. It would be appropriate to also consider undertaking sensitivity analysis when the linearity assumptions are not met. Considering the nature of the study, distribution used for simulation is supposed to the same as one applied using the data and this is not supposed to be included as a limitation. The analyses codes for both simulation and observational analyses should be made available for reproducibility purposes.

**Authors: We have deeply modified the manuscript. Now the simulation study is based on an generative cumulative logistic regression model. In addition, we propose a second simulative scenario in presence of model miss-specification.**

2) Authors need to present a DAGs for the COVID-19 observation study.

**Authors: We have added a new DAG Figure for the COVID-19 study (Figure 3).**

3) Make it clear whether survey weights were included in the PS model when computing weights?

**Authors: To increase the generalizability of the results we included in the PS model (GBM) and in the regression model (CLM) the sampling weights. We have added a sentence in the method section to explain sampling characteristics.**

Minor comments

Page 11 lines 63 -64 are not clear. Was it supposed to read “...characteristics that influence both the probability of the exposure and the outcome”?

**Authors: The sentence has been revised accordingly.**

In Table 3, summary of outcome variables (i.e. adherence to protective behaviour) by vaccination are not presented.

**Authors: The outcome variables are already present. We have highlighted those using horizontal lines.**

Page 26 line 347. State in which circumstances is PS advantageous.

**Authors: We have added several circumstances for which PS is advantageous (see the method section).**
