# Supplementary material for: Impact of COVID-19 vaccination on preventive behavior: The importance of confounder adjustment in observational studies
Source: PLoS One. 2024 Nov 25;19(11):e0313117. doi: 10.1371/journal.pone.0313117 (PMC11588266; doi:10.1371/journal.pone.0313117)
Supplement: Attachment — Submitted filename: reviewers_reply2_def.docx [file pone.0313117.s005.docx]

**Editor**

Thank you for submitting your manuscript to PLOS ONE. After careful consideration, we feel that it has merit but does not fully meet PLOS ONE’s publication criteria as it currently stands. Therefore, we invite you to submit a revised version of the manuscript that addresses the points raised during the review process.

In agreement with reviewer 1, I think the manuscript needs to address in detail their comments in its next revision in order to be accepted.  I agree that the intended contribution(s) of the manuscript are unclear. I suggest focusing on one clear contribution and making it very strong instead on trying to tackle three different types of contributions and not being convincing enough in neither. This will require rewriting some parts of your paper as well as possibly changing its current title, because in it is current form I think it is misleading as it suggest a review or a didactical paper about the specific methodology.

You are asked to addressed all reviewer 1's comments in detail.

**Authors:** **Thank you for your constructive feedback. We acknowledge the need to clarify the manuscript's contribution and we have focused on strengthening a single, clear contribution. We agree that the current approach, which attempts to address multiple contributions, lacks the necessary focus and impact. To address this, we have now revised the manuscript as a substantial contribution to the literature related to the impact of COVID-19 vaccine on behavioral preventive measures considering different adjusting methods. Additionally, we have revised the title to better reflect the manuscript's content.**

**At this end, we had addressed all of Reviewer 1's comments in detail in our review.**

**Reviewer #1: Review for PLOS ONE, manuscript PONE-D-24-03437_R1**

Biased Findings without Adjusting Methods in Observational Studies: Can Vaccination Status Influence Uptake of Preventive Measures against COVID-19?

Thank you for the ability to review this revised manuscript. The authors have clearly put a lot of effort into incorporating the feedback, and in parts, the manuscript sees some big improvements. At the same time, these changes have, in my opinion, not adequately addressed my major concern of the previous review, which is that the added value of this manuscript in relation to the vast (introductory) literature on causal inference methods is unclear to me. I will elaborate on this below by commenting on this manuscript’s statistical contribution to the literature, didactical contribution to the literature, and substantive contribution to the literature. I end with a list of further critical comments that I wrote up while reading the manuscript. I hope the authors find these comments useful for the improvement of their manuscript.

First, as a statistical contribution, the simulations are simply too limited, and focus on scenarios that have been previously investigated by other researchers as well. For example, in line 156 it is written: “A more comprehensive evaluation of different adjustment strategies in different simulation settings can be found in Schafer and Kang [28].”. Actually, Schafer and Kang (2008) did also include inverse probability weighting in their simulations, and this, in addition to the other simulation studies into IPW estimation that are references in the reference list of the manuscript, makes one wonder what the added-value is of these simulations? This is not clear in the current manuscript. Furthermore, the topic of double adjustment (i.e., as in Method D) has also already been extensively studied in Nguyen et al. (2017, doi: 10.1186/s12874-017-0338-0).

Second, as a didactical contribution, the manuscript falls short. My main concern here is that there are many different terms being used, but that the authors do not clearly describe how these are all related? I discuss some of these issues below in my list of further comments, but just a few examples: The authors mention the ignorability assumption, but do not explain that this (strong ignorability) is actually the combination of the exchangeability and positivity; the authors also mention assessing overlap in covariates, but do not explain that it is related to assessing the positivity assumptions; and the authors mention in line 116 the assumption of “independence” (I assume the authors refer to independence of residuals in a statistical model here), but then do not explain how this relates to exchangeability. In summary, the presentation of the assumptions that these methods rely on is simply unstructured, and therefore unclear for novices to IPW-based methods. It also again begs the question what is new that this manuscript brings to the table, especially compared to didactical introductions to IPW-estimation by the likes of Noah Greifer (<https://ngreifer.github.io/WeightIt/articles/estimating-effects.html>) and Naimi, Cole, and Kennedy (2017).

Third, as a substantive contribution to the literature, the manuscript also makes too many shortcuts (i.e., many important details are unexplained). For example:
- Are the sampling weights still valid when only a selection of the sample was used?;
- Why does the L.O.V.E. plot in Table S1 not all the age classes, educational levels, employment statuses, etc. (shouldn’t these categorical covariates have dummies, which are used for weighting?);
- Why were standard Wald confidence intervals used when it is recommended to use boostrapping to take the uncertainty of creating the weights into account (see Greifer, 2024, at <https://ngreifer.github.io/WeightIt/articles/estimating-effects.html#estimating-standard-errors-and-confidence-intervals>).
- Why was only a subsect of covariates selected for adjustment, as implied on line 318?

All in all, it is unclear to me what this manuscript is supposed to be. The further comments below also list some statements in the manuscript which I believe to be false (or unnuanced), and concerns regarding the validity of the simulation study results. All of these comments combined make me conclude that the manuscript is not ready for publication yet.

**Authors: Thank you for your detailed review and insightful comments. We appreciate the opportunity to refine our manuscript based on your feedback. We believe that the third option, making a substantive contribution to the literature, is our primary goal. We acknowledge that the current form of the manuscript raised concerns about the clarity and value of our manuscript. We hope the revised manuscript better articulates the novel aspects and significance of our work. Specifically, we have clarified that the added value of our simulations and methodological parts is to support the interpretation of the results related to COVID-19 vaccine data. In particular, we have provided a more detailed explanation of our decisions in relation to the substantive contribution to the literature.**

- **The sampling weights remain valid even if a portion of the data was selected, as our focus was on ensuring internal validity. Achieving external validity in this context is challenging due to the relative selection influenced by factors such as the pandemic dynamics, government policies, and population characteristics, that may change in future.**
- **For calculating the Standardized Mean Differences (SMD) for categorical variables, we employed the method proposed by Yang and Dalton (2012) (**[**link to the paper**](https://citeseerx.ist.psu.edu/document?repid=rep1&type=pdf&doi=6cf4bd36ca4c90006a5d6563f646a391c255581b)**).**
- **We have opted to use Wald confidence intervals solely for covariate adjustment, while bootstrap confidence intervals are now being employed for propensity score methods.**
- **We selected a subset of covariates for adjustment based on the original dataset, which contained a large amount of information not all relevant to this study. We focused on variables that are potential confounders in relation to the behavioral outcomes and the vaccination status.**

**To better reflect our change in the nature of the paper, we have also changed the title of the manuscript. In addition, we have strengthened the methodological part following your comments regarding the statistical and didactic contributions. All the sections has been deeply revised accordingly.**

Further comments:

- In general, there are many spelling mistakes and grammatically awkward sentences. For example, frequently the word “causal” is written as “casual”, and Figure 3 contains the word “counfounder” rather than “confounder”.

**Authors: We have carefully checked the document with the help of a native language speaker. We hope to have fully addressed the reported mistakes and the grammatical issues.**

- L. 45. “(…) the assumption of ignorability guarantees the unbiasedness of results (…)”. While this is a necessary assumption, unbiasedness is not guaranteed per se. If you have a continuous exposure and wrongly assume the exposure-outcome relationship is linear, then you can still have biased results (due to model misspecification) even when ignorability holds.

**Authors: We have rephrased the sentence in order to be more accurate in explaining the concept. In particular, in RCT we can also comparing the outcome without a regression model (so misspecification is not a problem), but just calculating the average of the outcome of the treated and untreated group since ignorability in general guarantees the comparability the results between the two groups without any further adjustment.**

- L 53. Shouldn’t it be “(…) Y1 and Y0 are the potential outcomes (…)”?

**Authors: We have corrected the sentence.**

- L. 55. “This does not apply to observational studies.” Disagree, it does apply also in observational studies, as this is just the definition of the effect of interest. However, you cannot simply take the means of both groups to estimate both effects, so the way to identify and estimate the estimand is different, but the estimand can still be defined as the ATE.

**Authors: We agree that the sentence can be unclear. We have rephrased the sentence in order to report as ATE can be estimated as well as by observational studies.**

- L 57. “preferred” compared to what? Furthermore, alternative modeling approaches, like IPW-estimation, can also "(…) quantify effects related to the dependent variable while controlling for a limited set of covariates.", so why is this characteristic of regression models an advantage per se?

**Authors: We have modified the sentence dropping the comparative verb.**

- L. 61. Not true, as the presence of multiple confounders is not necessarily a problem for outcome regression methods. Furthermore, model misspecification can also occur in IPW methods (when your exposure model is misspecified), so I don’t understand the point being made here.

**Authors: The sentence has been modified considering other issues in covariate adjustment as collinearity, over bias adjustment, and data sparsity.**

- L. 68. Does the target audience of this paper know what is meant by "overlapping distribution of covariates"?

**Authors: We have rephrased the sentence to make the concept clearer to a broader audience.**

- L. 70-76. First, the authors introduce propensity scores. Then, in lines 70 to 76, the authors talk about stratification based on individual covariates. This seems like a weird shift? Wouldn’t you stratify based on the propensity score? Furthermore, in the lines 70 to 73, it is mentioned that we need to match for confounders, and then in line 74 it is said that we need to additionally consider “(…) characteristics that influence both the probability of the exposure and outcome (…)”. How are these characteristics in line 74 different from the confounders in lines 70 to 73?

**Authors: we have modified the first sentence (70-73) while the second one (74-76) that presents the confounder meaning has been moved earlier.**

- Line 76-79. The sentence is unclear to me. What is also unclear is how reference 16 is relevant, which is about extending results from an RCT to a wider target population of interest, to stratification that is discussed earlier in this paragraph.

**Authors: In the new version of the introduction these lines have been removed.**

- Line 81. Although IPW estimation is the main focus of the paper, it is only mentioned in line 81. This is confusing as matching is discussed earlier in the introduction, but actually not at all what the paper focusses on.

**Authors: We have now introduced the IPTW earlier in the Introduction section.**

- L. 110. What is a “likely” impact?

**Authors: We agree the word “likely” can be unclear. We have removed it from the sentence.**

- L. 112. Confounders are not just “thought to be responsible”, the ARE actually responsible for difference in characteristics between treated and untreated. Furthermore, should a confounder also impact the outcome, for it to be a confounder? This is missing from this sentence.

**Authors: We believe the meaning of a confounder is now well explained in the introduction section. This sentence has been dropped.**

- L. 114. What does “specification of several assumptions to be correctly identified” mean?

**Authors: we have connected this sentence with the second one in order to report in the meaning specification of several assumptions thereafter.**

- L. 116. Why is “no interaction with the treatment variable” a key assumption? As long as I include an interaction when there is one, then I can still estimate my ATE. Admittedly, the coefficient itself is not the ATE anymore, but you can still estimate the ATE with g-computation, which involves multiple regression analysis.

**Authors: We agree and we have removed “interaction with the treatment variable” from the list.**

- L. 136. Here three fundamental assumptions are discussed, but aren’t these the assumptions of exchangeability and positivity, discussed 1 sentence earlier?

**Authors: We have dropped the first two points of the list that are a consequence of exchangeability and positivity assumptions defined in the sentence earlier. We have kept the model (miss-) specification, the third point of the list.**

- Figure 2. It needs further explanation, because I have no idea what I am looking at.

**Authors: We have expanded this paragraph to better describe the purpose of the figure and the descriptive results derived from it.**

- L. 217. The statement that GBM provided more precises estimates seems unnuanced. Perhaps for the scenario with a quadratic effect of Z, but not for the scenario with only a linear effect. Also, are you worried about overfitting when using GBMs? Is that an issue?

**Authors: We have corrected the sentence to emphasize the superiority of flexible methods like GBM when the relationship between Z and X is not linear. The problem of overfitting is a real issue for GBM, there are several control parameters that can help to mitigate this effect. However, we adopt the default arguments of the function method_gbm of the R package WeightIt. We made some attempts and for our simulation condition the proposed setting works well.**

- L. 228. Given that IPTW-based method can be quite inefficient, it seems appropriate to also look at a measure of variability of the estimates. For example, the MSE?

**Authors: We have now calculated and reported the MSE for both the scenarios.**

- L. 231. I do not see how this statement is backed up by the results. Comparing n = 200 to n = 500, relative bias is lower for n = 500. However, then it increases again. Furthermore, what is the uncertainty around these ARebias estimates? I think they overlap, as the point estimates of ARebias are so close. In that case, you wouldn’t conclude that ARebias decreased with increasing sample size.

**Authors: We agree with the comment. In the light of new results, we have modified the sentence according to uncertainty of the standard deviation of ARebias.**

- L. 234. Why is there a heightened risk of bias in all cases, as all relationships have been correctly specified, right? (i.e., they are all linear)

**Authors: In the new simulation, this phenomenon disappears, especially for large sample sizes. For small sample sizes, it can be attributed to the limited information for adjusting the estimates and implies a positive bias as reported for method A (no adjustment).**

- L. 248. Why is there still bias for method C? Should GBM be able to handle the quadratic relationship between Z and exposure? If so, then balancing should work, right?

**Authors: In the new simulation, this phenomenon disappears. We have worked on the GBM parameters as reported in the paper of Elith et al. 2008 (10.1111/j.1365-2656.2008.01390.x)**

- L. 266. For the empirical example, wouldn’t you say you are more interested in the average treatment effect in the treated (the ATT) rather than the average treatment effect (ATE), generally?

**Authors: for the real data application the PS weights are now based on ATT. We have modify the results section accordingly.**

- L. 312. "(...) we can't directly assess (...)" or "(...) we can't asses the direct effect (...)". In case of the latter, that would be a weird statement, given that this manuscript is all about trying to estimate causal effects by using adjustment methods.

**Authors: We agree with the suggestion. We have modified the sentence using the second proposed version.**

- L. 409. Can you ever say the assumption of exchangeability was met? Usually, exchangeability is presented as an untestable assumption, which can simply be made more or less plausible through thorough discussion of the included (and not included) covariates.

**Authors: We agree with the comment. In our manuscript, we aim to make the assumption of exchangeability as plausible as possible by thoroughly discussing the large set of covariates we included in our analysis. While we cannot definitively claim that the assumption was met, we have taken steps to ensure that it is reasonable within the context of our study.**

Reviewer #2: Dear Editor,

Authors have adequately addressed both the major and minor comments. Overall, this study will contribute to the current literature (both methodological and substantively) about covariate adjustment and propensity score methods. They have also clearly demonstrated both the strength and limitations of the approaches they have used under different scenarios which was missing in the first draft. They have shared the analysis code though I would suggest they make it open access where someone does not need to request permission from authors to access. For example, as part of supplementary material. Therefore, having read the revised paper and response to reviewer comments response I recommend the paper to be accepted in the current form.

**Authors: We thank you for the suggestions and the appreciation of the revised manuscript.**
