# Supplementary material for: Impact of COVID-19 vaccination on preventive behavior: The importance of confounder adjustment in observational studies
Source: PLoS One. 2024 Nov 25;19(11):e0313117. doi: 10.1371/journal.pone.0313117 (PMC11588266; doi:10.1371/journal.pone.0313117)
Supplement: Attachment — Submitted filename: Response to reviewer_rev3.docx [file pone.0313117.s006.docx]

**Editor**

Thank you for submitting your manuscript to PLOS ONE. After careful consideration, we feel that it has merit but does not fully meet PLOS ONE’s publication criteria as it currently stands. Therefore, we invite you to submit a revised version of the manuscript that addresses the points raised during the review process.

In the next revision, please address the comments made by the new reviewer and consider incorporating the literature they mentioned.

**Authors: Thank you for the possibility to revise the manuscript. In the current version of the paper we have replied to all the reviewer’ comments, incorporating the suggested literature where appropriate.**

**Reviewer 3: Review for PLOS ONE, manuscript PONE-D-24-03437_R2**

Title: Impact of COVID-19 Vaccination on Preventive Behavior: The Importance of Confounder Adjustment in Observational Studies

The paper investigates effect of vaccination on preventive behaviors by controlling estimation biases through appropriate method. Further, simulations are conducted to evaluate the degree of biases under various settings. I believe, the results are convincing and valuable for understanding interaction relation between vaccination and preventive behaviors. However, basic information about PSM is not sufficiently provided. This study would contribute to the literature if some additional works have been appropriately done. Specific comments are as follows.

1. I would like to see the results in the first stage of Propensity score matching. That is, present the results for estimating propensity score (in appendix).

**Authors: The results of the first stage of propensity score weighting have been moved from the supplementary material to the results section (new Figure 3).**

2. How did authors set the caliper to Author should conduct estimations using pooled sample and then put the cross terms between gender dummy (for instance male dummy when reference group is female) and key variables. Cite the following paper and show how to set caliper.

Wang Y, Cai H, Li C, Jiang Z, Wang L, Song J, et al. (2013) Optimal Caliper Width for Propensity Score Matching of Three Treatment Groups: A Monte Carlo Study. PLoS ONE 8(12): e81045. https://doi.org/10.1371/journal.pone.0081045

**Authors: As we used a propensity score weighting procedure, we had not considered a caliper stage, as all observations are weighted. In practice, if we estimate a weight close to zero, we do not include that observation in the analysis. To the best of our knowledge, the use of caliper is mainly related to propensity score matching to avoid controls (or cases) that have a low degree of matching with cases (or controls) after matching. Regarding the second part of the comment, as suggested, we included the interaction terms between gender and three key variables (age class, educational level, COVID-19 risk perception and conspiracy score) in both the propensity score model and the ordinal cumulative regressions.**

3. Indicates the effective sample sizes by illustrating Figures. Authors should cite Endo (2022) and follow the style of Figure 4 of Endo (2022).

Endo A. ‘Not finding causal effect’ is not ‘finding no causal effect’ of school closure on COVID-19. F1000Research 2022, 11:456 <https://doi.org/10.12688/f1000research.111915.1>

**Authors: We have added the effective sample size in the new Figure 3 (ex Figure S1).**

4. The following works are closely related to the paper. From the theoretical viewpoint to consider moral hazard, Peltzman effects (1975) should be noted. Considering the incentive to engage in preventive behaviors against COVID 19, authors should cite the following papers.

Asri A, Asri V, Renerte B, Föllmi-Heusi F, Leuppi JD, Muser J, et al. Wearing a mask-For yourself or for others? Behavioral correlates of mask wearing among COVID-19 frontline workers. PLOS ONE. 2021;16 (7 July):e0253621. doi:10.1371/journal.pone.0253621.

Cato S, Iida T, Ishida K, Ito A, Katsumata H, McElwain KM, et al. Vaccination and altruism under the COVID-19 pandemic. Public Health Pract (Oxf). 2022;3:100225. doi:10.1016/j.puhip.2022.100225.

Cato S, Iida T, Ishida K, Ito A, McElwain KM, Shoji M. Social distancing as a public good under the COVID-19 pandemic. Public Health. 2020;188:51-3. doi:10.1016/j.puhe.2020.08.005.

Hossain MdE, Islam MdS, Rana MdJ, Amin MR, Rokonuzzaman M, Chakrobortty S, et al. Scaling the changes in lifestyle, attitude, and behavioral patterns among COVID-19 vaccinated people: Insights from Bangladesh. Hum Vaccin Immunother. 2022;18:2022920. doi:10.1080/21645515.2021.2022920.

Peltzman S. The effects of automobile safety regulation. J Pol Econ. 1975;83:677-725. doi:10.1086/260352.

Yamamura, E.　Tsutsui, Y., Kosaka, Y., Ohtake, F. 2023. Association between the COVID-19 vaccine and preventive behaviors: Panel data analysis from Japan. Vaccines, 11(4), 810; https://doi.org/10.3390/vaccines11040810

Yamamura, E.　Tsutsui, Y., Ohtake, F. 2024. The COVID-19 vaccination, preventive behaviours and pro-social motivation: Panel data analysis from Japan. 　 Humanities and Social Sciences Communications, 11, Article number: 476

**Authors: We thank the reviewer for his suggestions; we have now considered the theoretical viewpoint of moral hazard in explaining behavioral effect of COVID-19 vaccination, including some of the suggested references in the discussion section**

5. In Tables 1 and 2, I cannot find information about what number in parentheses for “ARebias” in columns A, B, C, and D. To take an example, in the first line, “A” shows 1.318 (1.032). Is “(1.032)” standard error? Authors should explain it in the footnote of Tables 1 and 2.

**Authors: We have included in the headings of Table 1 and 2 specifying that the value in parentheses is the standard error of the estimates.**

6. In the PDF file of the manuscript, there seems to be three Figures as Figure 2 because there are three pages. Puzzlingly I cannot find two of Figures 2 because of blank on two pages although I find the first Figure 2 (Bars of “A” and “B”). Further, Figure 1 and the first of Figure 2 are too simple to be meaningful. Probably, these are more sophisticated with more useful information. I do not know the reason of poor presentation and disappearance of these Figures. Please made PDF file again to appropriately demonstrate these Figures. After checking it, I would like to provide final evaluation.

**Authors: We are very sorry for this inconvenience. We have double-checked the figure files that are in PDF format. All figures are correctly reported in the proof version of the submission and we hope that the problem has now been resolved.**
